# Secretome of Dental Pulp-Derived Stem Cells Reduces Inflammation and Proliferation of Glioblastoma Cells by Deactivating Mapk-Akt Pathway

**DOI:** 10.54457/DR.202302006

**Published:** 2023-07-19

**Authors:** Prateeksha Prateeksha, Md Sariful Islam Howlader, Surajit Hansda, Prathyusha Naidu, Manjusri Das, Faten Abo-Aziza, Hiranmoy Das

**Affiliations:** 1 Department of Pharmaceutical Sciences, Jerry H. Hodge School of Pharmacy, Texas Tech University Health Sciences Center, Amarillo, Texas 79106, USA

**Keywords:** Dental pulp-derived stem cells secretome, Inflammation, Cell cycle, Cellular bioenergetics, P38 MAPK-AKT pathway

## Abstract

**Background:**

Dental pulp-derived stem cells (DPSC) is a promising therapy as they modulate the immune response, so we evaluated the inhibitory effect of DPSC secretome (DPSC^℗^) on the proliferation and inflammation in human glioblastoma (GBM) cells (U-87 MG) and elucidated the concomitant mechanisms involved.

**Methods:**

The U87-MG cells were cultured with DPSC^℗^ for 24 h and assessed the expression of inflammatory molecules using quantitative reverse transcription-polymerase chain reaction (qRT-PCR), generation of reactive oxygen species (ROS), and mitochondrial functionality using a seahorse flux analyzer. MTT (3-(4, 5-dimethylthiazolyl-2)-2, 5-diphenyltetrazolium bromide) assay and cell cycle analysis were performed to evaluate the proliferation and cell cycle. Finally, the protein levels were determined by western blot.

**Results:**

DPSC^℗^ reduced the inflammation and proliferation of U-87 MG cells by down-regulating the pro-inflammatory markers and up-regulating anti-inflammatory markers expressions through ROS-mediated signaling. Moreover, DPSC^℗^ significantly reduced the mitochondrial membrane potential (MMP) in the cells. The cellular bioenergetics revealed that all the parameters of oxygen consumption rate (OCAR) and the extracellular acidification rate (ECAR) were significantly decreased in the GBM cells after the addition of DPSC^℗^. Additionally, DPSC^℗^ decreased the GBM cell proliferation by arresting the cell cycle at the G1 phase through activation (phosphorylation) of checkpoint molecule CHK1. Furthermore, mechanistically, we found that the DPSC^℗^ impedes the phosphorylation of the mitogen-activated protein kinases (P38 MAPK) and protein kinase B (AKT) pathway.

**Conclusion:**

Our findings lend the first evidence of the inhibitory effects of DPSC^℗^ on proliferation and inflammation in GBM cells by altering the P38 MAPK-AKT pathway.

## Introduction

Glioblastoma (GBM) is the most prevalent tumor of the central nervous system (CNS). It originates from star-shaped glial cells, known as astrocytes, which regulate the synaptic function of nerve cells^[1]^. In the United States, GBM accounts for 14.5% of all CNS tumors and nearly half (48.6%) of all malignant CNS tumors, making it the most prevalent primary brain tumor^[2]^. As per World Health Organization (WHO), it is ranked as a grade 4 glioma, which is currently being treated by surgical resection, radiation therapy, and chemotherapy. The effectiveness of these therapies is limited due to the complexity of the disease and the presence of the blood-brain barrier (BBB), thereby the drugs are unable to penetrate the tumor located in the brain ^[3]^. Also, some treatment options cause various complications such as neurotoxicity, cognitive disturbance, and secondary malignancy. Thus, GBM patients exhibit an abysmal prognosis with a 1-year life expectancy rate of 37.2% and a 5-year life expectancy rate of 5.1%^[4]^. Therefore, significant advancements are warranted to develop effective and safe therapy.

GBM cells produce a variety of cytokines, including transforming growth factor-beta (TGF-β), interleukin-6 (IL-6), and tumor necrosis factor-alpha (TNF-α). These cytokines create an inflammatory milieu and amplify the tumor progression and invasion^[5,6]^. Thus, inflammation is the molecular signature of cancer progression, and it also lends resistance to GBM cells for chemotherapy and radiotherapy^[7]^. Targeting inflammation for GBM therapy has become an active area of research. Several anti-inflammatory commodities have been developed and examined, but these drugs have yet to be proven effective in clinical trials^[8]^. So, researchers are continuing to search for better effective therapy for GBM. Mesenchymal stem cells (MSC) are a cynosure for the scientific world due to their potential therapeutic applications in various diseases, including cancer, autoimmune disorders, and tissue damage caused by injury or degenerative diseases. MSC exert pleiotropic effects in pathophysiological conditions, including anti-inflammatory, anti-fibrotic, anti-apoptotic, etc. The immune suppression ability of MSC is especially important for clinical use because it’s co-transplantation reduces the chance of immune rejection by the host immune system^[9]^. Nevertheless, preclinical studies conferred contradictory results depicting both pro- and anti-cancer effects of MSC; therefore, their therapeutic potential for cancer treatment has been obstructed^[10]^.

MSC can be derived from various tissues, including adipose (fat) tissue, synovial fluid, amniotic fluid, bone marrow, placenta, umbilical cord tissue, and others through invasive and noninvasive approaches. Invasive procedures raise the risk of complications, such as bleeding, nerve injury, or damage to surrounding tissues, which can further limit the utility of MSC obtained through invasive procedures^[11]^. Interestingly, human dental pulp stem cells (DPSC) are acquired by noninvasive procedures from third molar teeth and their therapeutic potential resembles other kinds of MSC which allow them to treat debilitating diseases such as immunodeficiencies, neurological diseases, type 1 diabetes, and bone and cartilage disorders^[12–14]^. Strong evidence demonstrated that the culture supernatant of MSC (MSC^℗^) governs various cellular functions such as proliferation, migration, differentiation, and communication due to its cell secretome, which comprises a range of factors, including exosomes, cytokines, and growth factors^[15]^. Exosomes present in the MSC^℗^ contain a variety of biomolecules, including DNA, microRNA (miRNA), small interfering RNA (siRNA), long non-coding RNA (lncRNA), proteins, and lipids. These molecules are particularly important as therapeutic elements for tumor treatment^[16]^. Moreover, exosomes have been utilized as nanocarriers for drug and nucleic acid delivery^[17]^. The inherent anticancer activity of exosomes can enhance the effectiveness of drugs when used as a nanocarrier. Therefore, exosomes derived from MSC have gained significant attention as a promising avenue for the development of new and effective therapies^[18]^. However, a plethora of scientific studies has provided compelling evidence that the entire secretome, including exosomes, interacts with cancer cells and effectively inhibits their growth and migration^[19–21]^. As previously illustrated that MSC^℗^ decrease breast cancer cell growth and sensitize cancer cells to radiotherapy by downregulating the signal transducer and activator of transcription 3 (STAT3) signaling pathway^[22]^. We previously found that DPSC-secreted factors augment wound healing in diabetic mice by upregulating the SMAD signaling^[13]^, and suppressing osteoclastogenesis by secreting osteoprotegerin and inhibiting AKT signaling in monocytes^[12]^. It was reported that DPSC^℗^ inhibit viability and induces apoptosis in human colorectal cancer cells^[23]^. Also, DPSC has the ability to interact with biomaterial scaffolds and proliferate on them like a natural extracellular matrix^[24]^. Thus, DPSC^℗^ can be applied as an effective and practical cellular therapy for treating glioma. Here, we aimed to evaluate the anti-inflammatory and anti-proliferative effects of the DPSC^℗^ on human GBM, U-87 MG, and decipher the cornerstone of the molecular mechanism associated with its inhibitory effects. This study will highlight the therapeutic potential of DPSC^℗^ for combating the aggressiveness of glioma, and it could be used as adjuvant therapy in combination with other treatment modalities, such as chemotherapy or radiation therapy, in the future.

## Materials and Methods

### Materials

The human GBM cell line U-87 MG (#HTB-14^™^) was procured from ATCC. The antibiotic and antimycotic solutions (anti-anti, #15240), cDNA kit (#4387406), SYBR Green PCR master mix (#4309155), MitoSOX red compound (#M36008), RNase (#EN0531), protease and phosphatase inhibitors (#78441), ethanol (#UN1170) and glutamine (#25030081) were obtained from ThermoFischer Scientific, Waltham, MA, USA. The minimum essential medium eagle (MEME, #M8042), 2’,7’-dichlorodihydrofluorescein diacetate (DCFDA, #4091–99-0), Triton X-100 (#T8787), bovine serum albumin (BSA, #A7906), RIPA buffer (#20–188) and protein marker (#GERPN800E) were purchased from Millipore Sigma Aldrich Corporation, Burlington, MA, USA. Seahorse Mito Stress assay (#103015– 100), glycolysis stress assay (#103020–100), pyruvate (#103578–100), glutamine (#103579–100), glucose (#103577–100), Seahorse XF DMEM (#102353–100) were procured from Agilent, USA. ProLong Gold Antifade Mountant with 4’,6-diamidino-2-phenylindole (DAPI, #P36931), and propidium iodide (PI, #P3566) were purchased from Invitrogen, USA. The precision red advanced protein assay kit (#ADV02), and Roche Cell Proliferation Kit I (#11465007001) were obtained from Cytoskeleton Inc., Denver, CO, USA, and Roche Diagnostics, Basel, Switzerland, respectively. The nitrocellulose membrane (#1620115) and Laemmli buffer (#1610737) were purchased from Bio-Rad Laboratories, Hercules, CA, USA. Tris-buffered saline (TBS) with 0.1% Tween 20 detergent (TBST, #IBB-180) was procured from Boston Bioproducts, Milford, MA, USA. The fetal bovine serum (FBS, #PS-FB3) was obtained from Peak Serum, Wellington, CO, USA. The peroxidase (HRP) conjugated secondary antibodies (#7074S, #7076S), and chemiluminescence substrate (#RPN2232) were obtained from Cell Signaling, USA, and Amersham Pharmacia Biotechnology, Amersham, UK, respectively. The Instruments, XF24 Extracellular Flux Analyzer, CFX96 Real-Time System, super-resolution confocal microscope, FACSVerse flow cytometer, and microplate reader were purchased from Seahorse Bioscience (Billerica, MA, USA), Bio-Rad Laboratories (Hercules, USA), Leica Stellaris 8 Falcon STED (Germany), BD Biosciences (East Rutherford, NJ, USA), and Synergy 2, BioTeK Instruments Inc (Winooski, USA), respectively.

### Cell lines and cell culture

The human GBM cell line U-87 MG was grown in Dulbecco’s Modified Eagle Medium (DMEM) comprising 10% FBS and 1% anti-anti. Primary DPSC were isolated from the third molar teeth of healthy adolescent donors, as reported earlier^[25]^. Homogeneity of the isolated population was also confirmed by assessing the expression of MSC-specific surface markers (CD73, CD90, and CD105) using flow cytometry^[25]^.

### Collection of DPSC secretome (DPSC^℗^)

Primary DPSC (passage 2 - passage 7) were grown in MEME with 20% FBS, 1% anti-anti and 1% glutamine on a 60 mm cell culture plate at the density of 1 × 10^6^ for 36 h in a humidified incubator maintain the temperature 37 °C and an atmosphere of 5% CO_2_. The medium is then replaced by the fresh DMEM culture medium, which is used for the proliferation of U-87 MG cells and kept in a CO_2_ incubator for 36 h. The DPSC^℗^ was collected and used for subsequent experiments.

### qRT-PCR analysis

GBM cells (U-87 MG) were cultured in a 6-well plate overnight at 1 × 10^5^ cells/well in a humidified incubator maintaining the temperature at 37 °C and the atmosphere of 5% CO_2_. Day after the cell seeding, the medium was exchanged with fresh DMEM and DPSC^℗^ in the control and treatment groups, respectively and re-incubated for 24 h. The U-87 MG cells were rinsed with phosphate buffer saline (1 × PBS) and harvested in TRIzol reagent for RNA isolation. The quantity and quality of isolated RNA were determined by a NanoDrop 8000 (Thermo Scientific). The reverse transcription for each sample was performed using a cDNA kit following the manufacturer’s instructions. Next, the SYBR Green PCR master mix was used to amplify the complementary DNA (cDNA). The qRT-PCR was performed using the CFX96 Real-Time System. The cycling parameters were kept the same as instructed by the manufacturer. The calculated Ct values (threshold cycle) of target genes were normalized with internal control, β-Actin, and relative target transcripts expression was expressed as fold change. All primer sets of studied target genes are included in the supplementary Fig. 1.

### Cellular ROS analysis

The DCFDA was used to detect the intracellular ROS generation. Non-fluorescent DCFDA binds with intracellular ROS and is converted into a fluorescence probe. In short, U-87 MG cells were seeded on the coverslip and placed in a 6-well plate with/without DPSC^℗^ at the density of 1 × 10^5^ cells/well and incubated overnight in a humidified incubator maintaining the temperature at 37 °C and an atmosphere of 5% CO_2_. The next day, the medium is replaced by DPSC^℗^ and re-incubated for 24 h keeping controls using the same culture medium. The cells were then incubated in 5 μM of DCFDA for 20 min at 37 °C followed by washing 3 times with 1 × PBS and after that, coverslips were fixed on glass slides using ProLong Gold Antifade Mountant with DAPI. The images of each group of cells were captured using a super-resolution confocal microscope at excitation/emission of 495/529 nm, and LAS X image analysis software was employed to analyze the images.

### Mitochondrial ROS analysis

The mitochondrial ROS production was determined using the MitoSOX red compound following our published protocols^[26–28]^. Non-fluorescence MitoSOX red oxidizes with free radicals of mitochondrial superoxide and produces red fluorescence. In brief, the cells were grown with/without DPSC^℗^ for 24 h, as discussed above. The cells were incubated in 1 mL of Hanks’ balanced salt solution (HBSS) containing 5 μM of the MitoSOX red for 15 min at 37 °C. After washing 3 times with 1 × PBS, the images of each group of cells were captured using a super-resolution confocal microscope at excitation and emission of 510/588 nm, and LAS X image analysis software was employed to analyze the images.

### Determination of mitochondrial membrane potential (MMP)

The mitochondrial membrane potentials (MMP) were determined by JC-1 staining as described previously^[29,30]^; experiments were set as described above. The DPSC^℗^-treated cells and untreated cells were incubated in 5 μM of JC-1 dye for 15 min at 37 °C. After 3 times rinsing with 1 × PBS, coverslips were fixed on the glass slides using a mountant containing DAPI. The images of each group of cells were captured using a super-resolution confocal microscope at the emission of 525 nm and 590, and LAS X image analysis software was employed to analyze the images. The ratio of the mean fluorescence intensity of the JC1 monomers (depolarized mitochondria) to JC1 aggregates (polarized mitochondria) indicates the MMP of cells.

### Determination of cellular bioenergetics

To determine the effect of DPSC^℗^ on the cellular bioenergetics, the oxygen consumption rate (OCR) and extracellular acidification rate (ECAR) were performed by Seahorse Mito Stress assay, and glycolysis stress assay using an XF24 Extracellular Flux Analyzer. In short, the cells (2 × 10^4^) were seeded in XFe24-well microplates overnight and treated with DPSC^℗^ for 24 h in a CO_2_ incubator at 37 °C. Before the day of seahorse analysis, the sensor cartridge was hydrated in Seahorse XF calibrant at 37 °C in a non-CO_2_ incubator overnight. The next day, the culture media of the cell culture plate was replaced by 500 μL of Seahorse assay medium prepared immediately with Seahorse XF DMEM comprising, glutamine, pyruvate, and glucose and placed in a non-CO_2_ incubator at 37 °C for one hour before running the assay. However, no pyruvate and glutamate were supplemented in the Seahorse assay medium when ECAR was performed. For determination of OCR, the component of MitoStress assay, including 2 μM of complex III inhibitor rotenone, and antimycin A, 1 μM of un-coupler carbonyl cyanide-4-(trifluoromethoxy) phenylhydrazone, and 1.5 μM of ATP synthase inhibitor oligomycin was loaded to XFe24 sensor cartridges plate from port C to A and incubated for 20 min in a non-CO_2_ incubator. Whereas, For ECAR analysis, the component of glycolysis stress assay, including glycolytic inhibitor 2-deoxy-D-glucose (2-DG, 50 mM), oligomycin (1 μM), and D-glucose (10 mM) were injected accordingly. After calibration with the prepared XFe24 sensor cartridges plate, the XFe 24-well cell culture microplates were placed in Flux Analyzer; the Seahorse MitoStress assay and glycolysis assay were run separately, and the OCR and ECAR values were estimated using the standard and unmodified protocol after normalization of values to total cell protein by Seahorse Wave software 2.6.1.

### Determination of cell proliferation

To determine the anti-proliferative effect of DPSC^℗^ on U-87 MG cells, the Roche Cell Proliferation Kit I was employed, and results were expressed as the percentage viability calculated by well-established formula (20). In short, 1 × 10^4^ U-87 MG cells were seeded in a well of a 96-well plate and incubated overnight at 37 °C in a 5% CO_2_ incubator. Day after the cell seeding, the media were exchanged with fresh DMEM and DPSC^℗^ in the control and experimental groups, respectively, and re-incubated for 24 h in a humidified incubator maintaining the temperature at 37 °C and the atmosphere of 5% CO_2._ Afterward 20 μL of MTT reagent was added to each well and incubated for 4h in the same culture condition. The formed formazan crystal was dissolved in the 200 μL of solubilizing buffer, and absorbance was recorded using a microplate reader.

### Analysis of cell cycle phases

Approximately 1 × 10^5^ U-87 MG cells/well were cultured in a 6-well plate and incubated with DPSC^℗^ keeping controls using the same culture medium for 24 h in a humidified incubator maintaining the temperature at 37 °C and the atmosphere of 5% CO_2_. The cells were rinsed 3 times with 1xPBS and harvested in a microcentrifuge tube by centrifugation. After fixing the cells overnight in 70% ethanol at –20 °C. the cells were then re-centrifuged and then suspended in 1xPBS containing 0.1 mg/mL of RNase, 100 μg/mL of PI, and 0.1% of Triton X-100 in a dark room for 20 min at 37 °C and were analyzed by FACSVerse flow cytometer. The distribution of the percent cell population in different phases of the cell cycle in each experimental condition was estimated using a flow cytometer inbuilt software (BD FAC-SSuite).

### Western blot analysis

The pre-cooled RIPA buffer comprising the protease and phosphatase inhibitors was used to extract the protein from each group of cells by following the method used previously^[31]^. The quantification of total cell protein concentration was done with a precision red advanced protein assay kit. The volume of each sample for 40 μg of protein was normalized and mixed with Laemmli buffer followed by separated on 10% SDS-PAGE gels. The separated protein on the gel was further transferred onto a nitrocellulose membrane by supplying the constant voltage of 30 mV at 4 °C. Afterward, the blocking solution was prepared in TBST and 5% BSA and added to the nitrocellulose membrane for 2 h at room temperature. The blocked membranes were incubated with primary antibody overnight at 4 °C followed by 3 times washing with TBST for 10 min each. The next day, the primary antibody probed nitrocellulose membranes were incubated in species-specific horseradish peroxidase (HRP) conjugated secondary antibodies for 2 h at room temperature. The washed membranes were then exposed to an enhanced chemiluminescence substrate. The protein bands were visualized, and band intensity was normalized to the internal control, GAPDH using Image J software (National Institutes of Health, Bethesda, MD, USA). Primary antibodies used in this study are mentioned in Supplementary Fig. 2.

### Molecular docking analysis

The molecular interaction of DPSC^℗^, namely, brain-derived neurotropic factors (BDNF) and glial cell-derived neurotropic factors (GDNF) with P38 MAPK and AKT was ensured using methods established in the literature^[32]^. The 3D crystal structure of BDNF, GDNF, human P38 MAPK, and AKT was obtained from the protein data bank (PDB) (https://www.rcsb.org/) with the PDBID 1BND, 2V5E, 1WBV, and 6HHG respectively. Then, we selected the appropriate protein chain of each molecule and prepared them for docking analysis using Swiss-Pdb Viewer^[33]^. The ClusPro 2.0 server (https://cluspro.bu.edu/) works on the PIPER algorithm and was applied to determine the interaction between BDNF and GDNF, and P38 MAPK and AKT. ClusPro 2.0 server yields the top ten refined models based on high binding affinity^[32]^. We analyzed all models to select the best model based on the least conformational changes, lower energy docked complex, and higher participation of pocket residues using PyMOL^[34]^.

### Statistical Analysis

All results are expressed as mean ± standard error of the mean (SEM). The differences between the two independent groups were calculated by a Student’s t-test using Graph Pad Prism 5.0 (Graph Pad Software, San Diego, CA, USA).

## Results

### Effect of DPSC^℗^ on inflammation in GBM cells

**Fig. 1.**
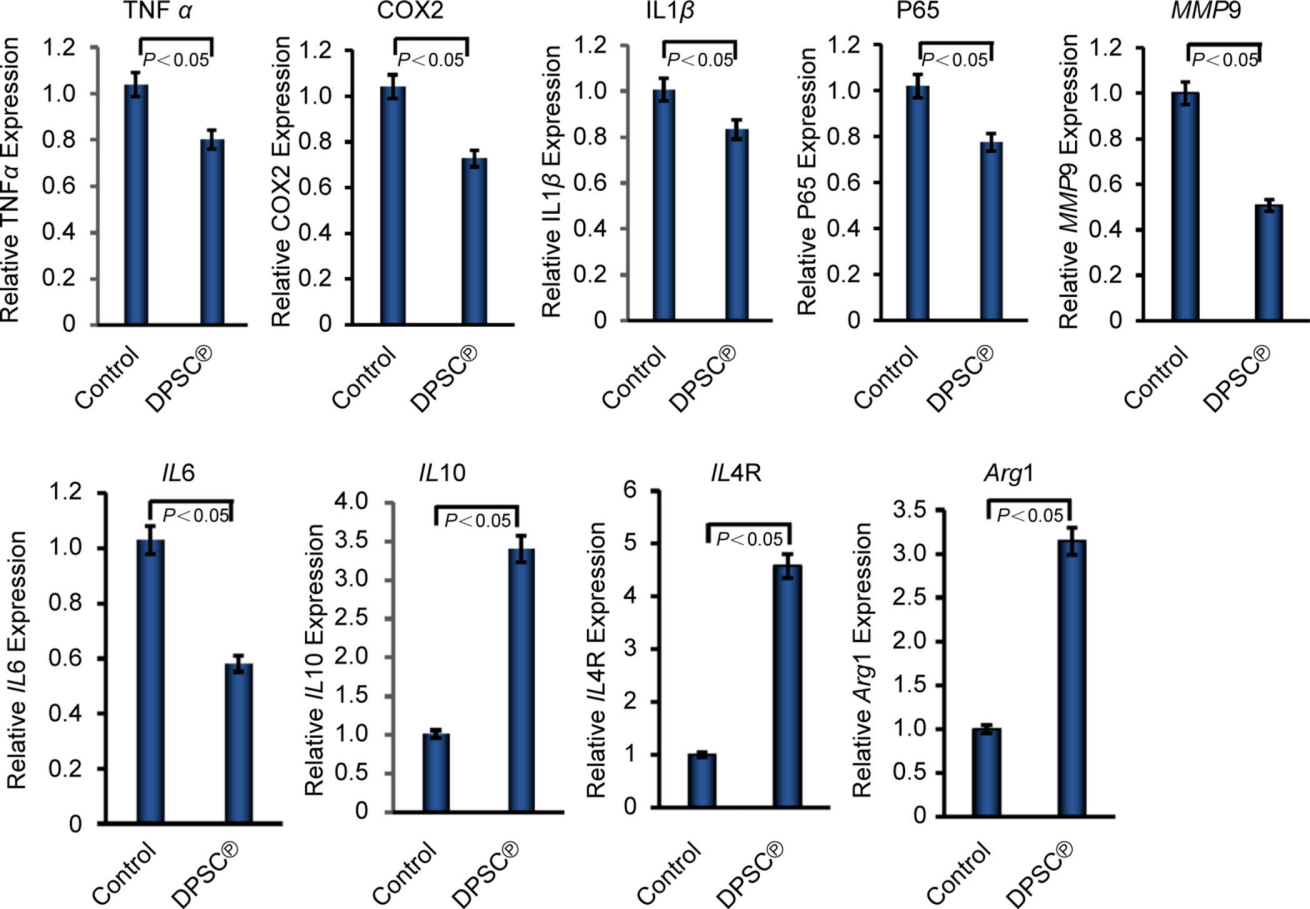
DPSC^℗^ reduced inflammatory and induced anti-inflammatory molecules in GBM cells. qRT-PCR analysis of proinflammatory marker molecules including TNFα, COX2, IL1β, p65, MMP9, and IL6; and anti-inflammatory marker molecules including IL10, IL4R, and Arg1 were determined in U-87 MG cells in the absence or presence of DPSC^℗^ during the culture of cells for 24 h.

The DPSC^℗^ reduced the inflammation in several severe pathological conditions; therefore, to ensure whether DPSC^℗^ reduced inflammation in GBM, we investigated the effect of DPSC^℗^ on pro- and anti-inflammatory molecules in U-87 MG cells using qRT-PCR analysis. We obtained that the expression level of pro-inflammatory markers, including TNFα, cyclooxygenase 2 (COX2), IL1β, NF-kappa-B p65 (p65), matrix metalloproteinases 9 (MMP9), and IL6 significantly (P < 0.05) decreased by 0.80, 0.73, 0.83, 0.775, 0.506, and 0.581, respectively in U-87 MG cells in presence of DPSC^℗^ compared to control where DPSC^℗^ were absent (Fig. 1). As expected, all examined anti-inflammatory marker molecules such as IL10, interleukin 4 receptor (IL4R), and arginase 1 (Arg1), were significantly (P < 0.05) elevated in the presence of DPSC^℗^ (Fig. 1). These data suggest that DPSC^℗^ possesses anti-inflammatory efficiency that may aid in combating the aggressiveness of GBM cells.

### Effect of DPSC^℗^ on the generation of intracellular and mitochondrial ROS in GBM cells

**Fig. 2.**
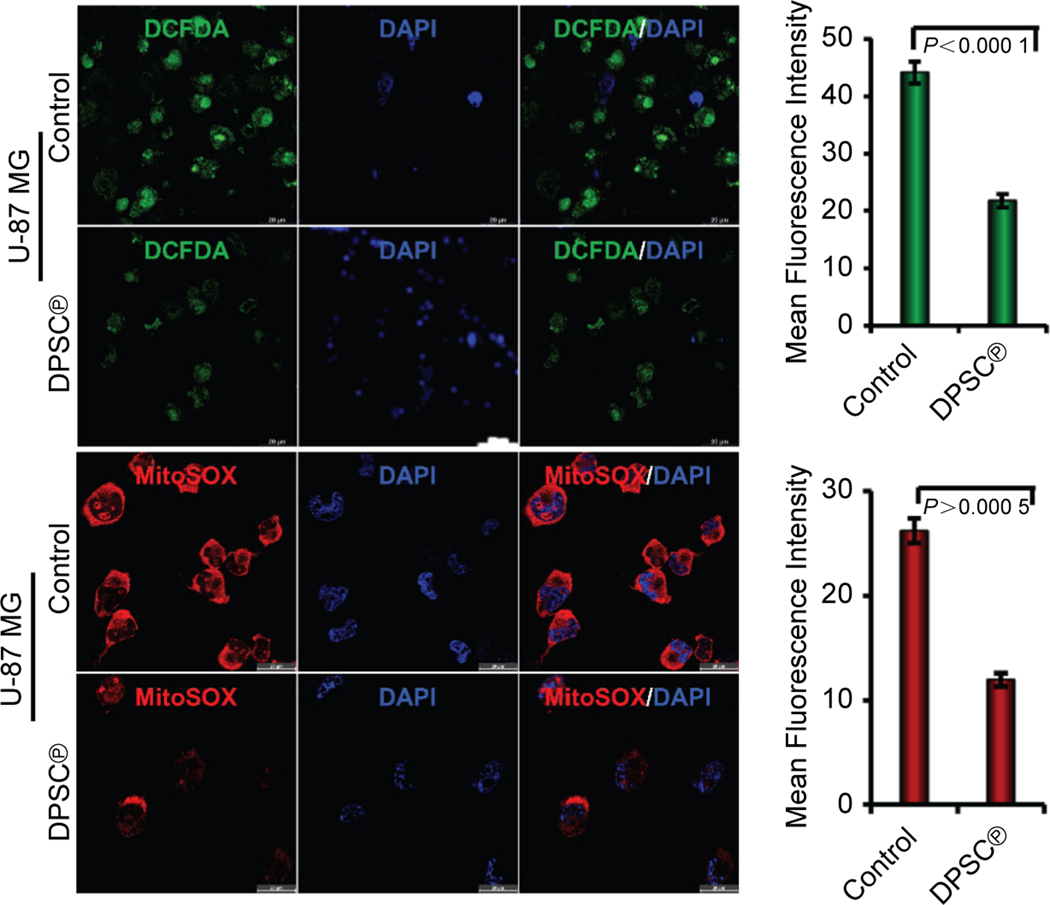
DPSC^℗^ reduced intracellular and mitochondrial ROS in GBM cells. Upper panels represent DCFDA staining of U-87 MG cells in the absence or presence of DPSC^℗^ during the culture of cells for 24 h. The adjacent graph represents the quantification of the mean fluorescence intensity of the same (n = 6, Scale bar, 20 μm). Lower panels represent MitoSOX staining of U-87 MG cells in the absence or presence of DPSC^℗^ during the culture of cells for 24 h. The adjacent graph represents the quantification of the mean fluorescence intensity of the same (n = 6, Scale bar, 20 μm).

Next, we evaluated the effect of DPSC^℗^ on total intracellular and mitochondrial ROS generation using the DCFDA and MitoSOX red staining and found that the U-87 MG cells displayed a higher level of total intracellular ROS in the absence of DPSC^℗^, indicating the higher metabolic activity of glioma cells. However, a significant decrease in the mean fluorescence intensity of DCFDA was observed in the cells after the addition of DPSC^℗^ (Fig. 2, upper panels). Similar to DCFDA staining, U-87 MG cells showed an elevated level of ROS, but it was remarkably decreased in the presence of DPSC^℗^ (Fig. 2, lower panels). These data were in the track with the anti-inflammatory effect of DPSC^℗^.

### Effect of DPSC^℗^ on mitochondrial membrane potential (MMP) in GBM cells

**Fig. 3.**
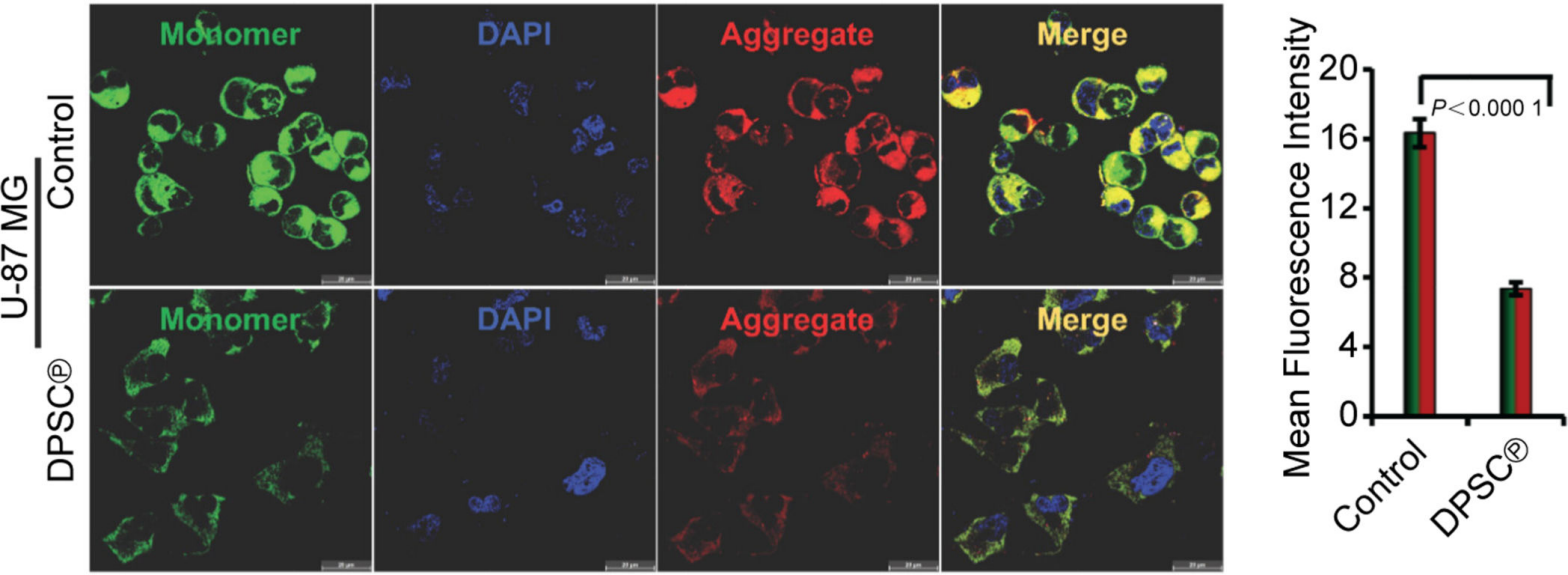
DPSC^℗^ reduced mitochondrial membrane potential in GBM cells. The left panels represent JC1 staining of U-87 MG cells in the absence or presence of DPSC^℗^ during the culture of cells for 24 h. The right graph represents the quantification of the mean fluorescence intensity of the same (n = 6, Scale bar, 20 μm).

Mitochondrial dysfunction is directly linked with pathogenesis in several disease conditions^[27, 35–38]^; therefore, we wanted to evaluate the effect of DPSC^℗^ on the MMP in U-87 MG cells using JC1 staining. JC1 accumulates in polarized mitochondria and emits red fluorescence, while JC1 remains in monomer form in depolarized mitochondria and emits green fluorescence. The decrease in the red/green fluorescence ratio indicates MMP depolarization. As illustrated in Fig. 3, the addition of DPSC^℗^ remarkably decreased the MMP in U-87 MG cells. The obtained results demonstrate that U-87 MG cells had high MMP, but in the presence of DPSC^℗^, the mitochondria switch towards depolarization and exhibit low MMP. These findings indicate that the addition of DPSC^℗^ in U-87 MG significantly reversed the mitochondrial dysfunction, leading to a decrease in cellular energy production and cell proliferation.

### Determine the effect of DPSC^℗^ on cellular bioenergetics in GBM cells

**Fig. 4.**
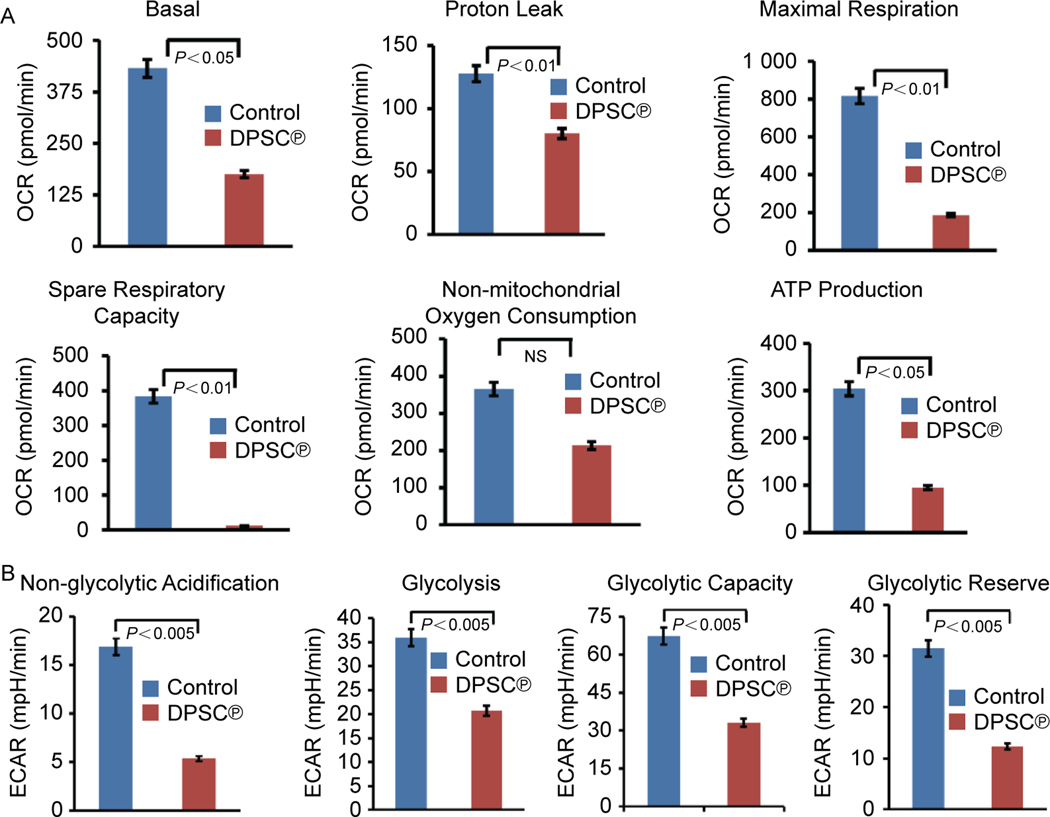
DPSC^℗^ reduced mitochondrial function in GBM cells. A. Seahorse flux analysis of oxygen consumption rate (OCR) of U-87 MG cells in the absence or presence of DPSC^℗^ during the culture of cells for 24 h. B. Seahorse flux analysis of extracellular acidification rate (ECAR) of U-87 MG cells in the absence or presence of DPSC^℗^ during the culture of cells for 24 h.

Next, we sought to determine the effect DPSC^℗^ on mitochondrial respiration and adenosine triphosphate (ATP) production in U-87 MG cells; therefore, we calculated the OCR and ECAR of U-87 MG cells in the presence/absence of DPSC^℗^ using an XF24 Extracellular Flux Analyzer. We observed a significant decrease in basal respiration, maximal respiration, proton leak, non-mitochondrial oxygen consumption, and respiration coupled to ATP production in the presence of DPSC^℗^ compared to control cells where DPSC^℗^ was absent. This result suggests that the DPSC^℗^ have a substantial impact on reducing the OCR. (Fig. 4A and Supplementary Fig. 3A). Similarly, all the parameters of ECAR, including non-glycolytic acidification, glycolysis, glycolytic capacity, and glycolytic reserve, were decreased in GBM cells cultured in the presence of DPSC^℗^ compared to control cells (Fig. 4B and Supplementary Fig. 3B). These data correlate with the aforementioned inhibitory effects of DPSC^℗^ on the aggressive proliferation or progression of GBM cells.

### Effect of DPSC^℗^ on cell proliferation and cell cycle in GBM cells

**Fig. 5.**
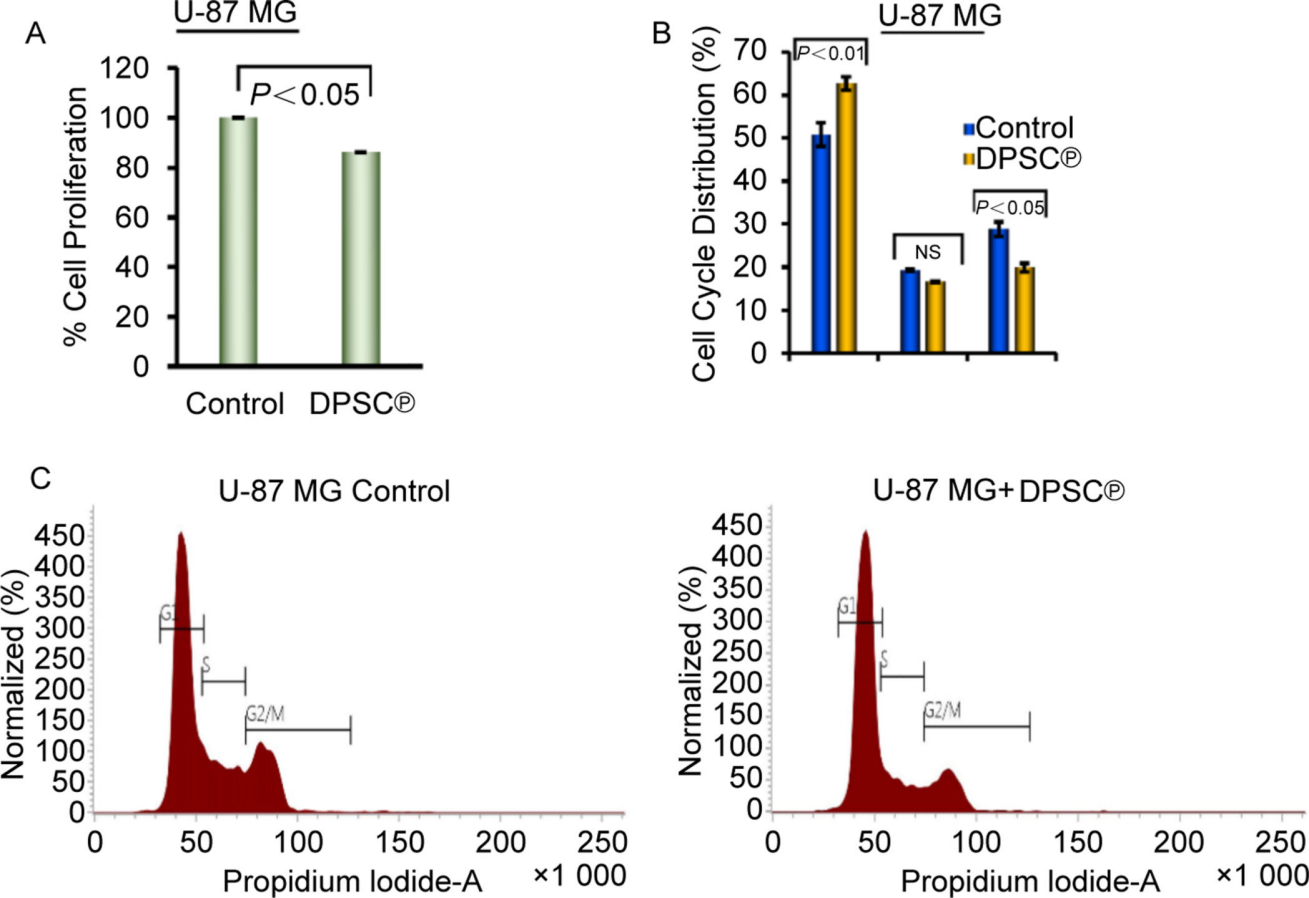
DPSC^℗^ reduced GBM cell proliferation by arresting the cell cycle. A. The cell proliferation of U-87 MG cells in the presence or absence of DPSC^℗^ during the culture of cells for 24 h. B. Quantitative graphical representation of percent population in each phase of the cell cycle in the absence or presence of DPSC^℗^ during the culture of U-87 MG cells for 24 h. C. Cell cycle histograms of U-87 MG cells in the absence or presence of DPSC^℗^ during the culture of cells for 24 h.

To evaluate the inhibitory effect of DPSC^℗^ on the proliferation of U-87 MG cells, an MTT assay was performed and obtained results were shown in Fig. 5A. Briefly, the cell proliferation of U-87 MG was decreased by up to 86 % after the addition of DPSC^℗^ and cultured for 24 h. This data indicates the DPSC^℗^ efficiently combats the aggressive proliferation of GBM cells. Next, we sought to examine how DPSC^℗^ regulates the cell cycle to slow down the uncontrolled proliferation of U-87 MG cells. The results of the cell cycle analysis were shown in Fig. 5B, C. The cell cycle population in the G1 phase, S phase, and G2/M phase were 50.76%, 19.25%, and 28.8%, respectively, in control U-87 MG cells. A significant increase in the cell population percentage in G1 (up to 62.60%) and a decrease in the G2/M phase (to 19.87%) was detected when U-87 MG cells were cultured in the presence of DPSC^℗^ for 24 h. No significant alteration was observed in the S phase (P < 0.05); however, the trend was towards the decreased number of populations after the addition of DPSC^℗^ to the GBM cells. These data clearly show that the DPSC^℗^ decreases the cell proliferation rate of U-87 MG cells by arresting the cell cycle at G0/G1 phase.

### Effect of DPSC^℗^ on molecular signaling pathways in GBM cells

**Fig. 6.**
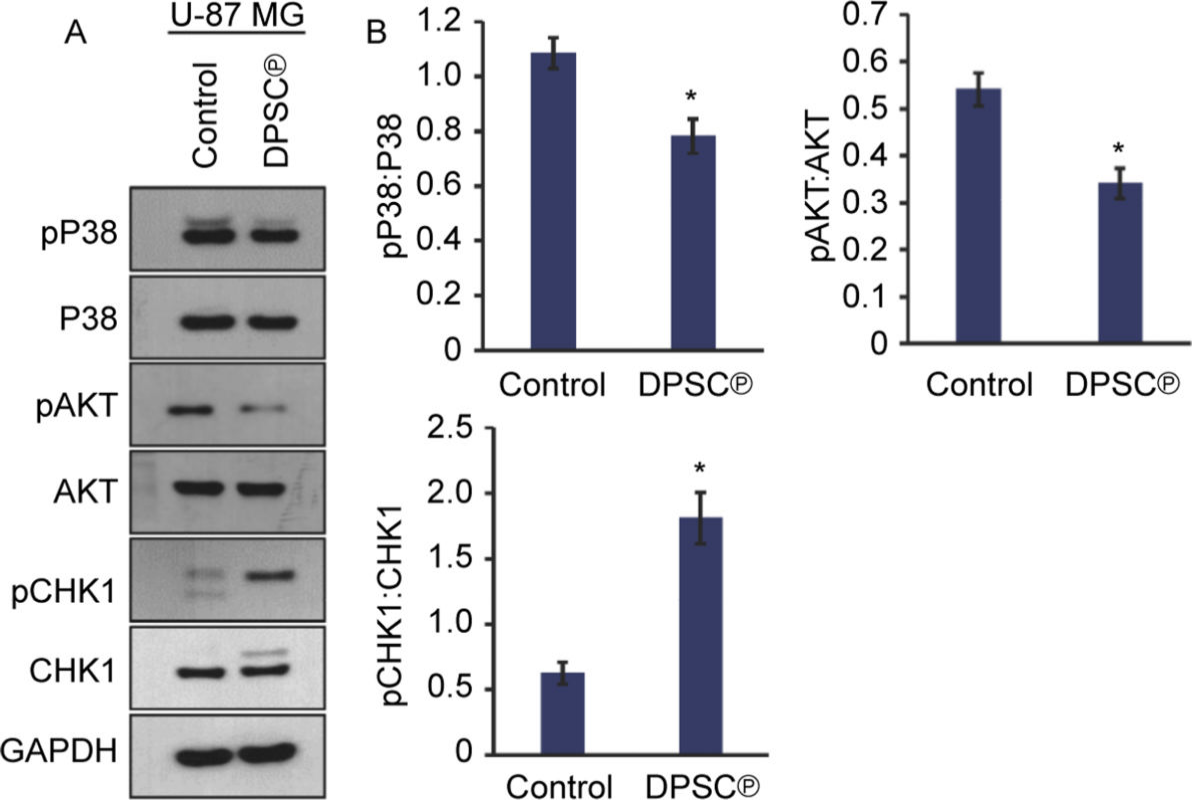
DPSC^℗^ reduced GBM cell proliferation by reducing activation of P38 MAPK and AKT pathways and activating checkpoint arrest pCHK1 molecule. A. The images of western blot analysis of pP38, P38, pAKT, AKT, pCHK1, and CHK1 keeping GAPDH as an internal control in U-87 MG cells in the absence or presence of DPSC^℗^ during the culture of cells for 24 h. B. The graphs represent the quantitative values of the analyzed proteins.

To elucidate the molecular mechanism associated with the anti-proliferative and anti-inflammatory effect of DPSC^℗^, we performed the western blot analysis investigating closely associated pathways and found that the activation of pP38 MAPK was significantly decreased in GBM cell lines after the addition of DPSC^℗^ during the culture of U-87 MG cells for 24 h. Furthermore, we found that a significantly downregulated the phosphorylation of AKT in U-87 MG cells after the addition of DPSC^℗^ during the culture of U-87 MG cells for 24 h (Fig. 6A, B and Supplementary Fig. 4). We were also interested to know how DPSC^℗^ affected the cell cycle and interestingly found that the hyperactivation (phosphorylation) of check point kinase 1 (CHK1), which is the molecular signature of checkpoint arrest for the cell cycle after the addition of DPSC^℗^ during the culture of U-87 MG cells for 24 h (Fig. 6).

### Defining in-depth regulatory mechanisms of molecular interactions for anti-proliferative and anti-inflammatory effects

**Fig. 7.**
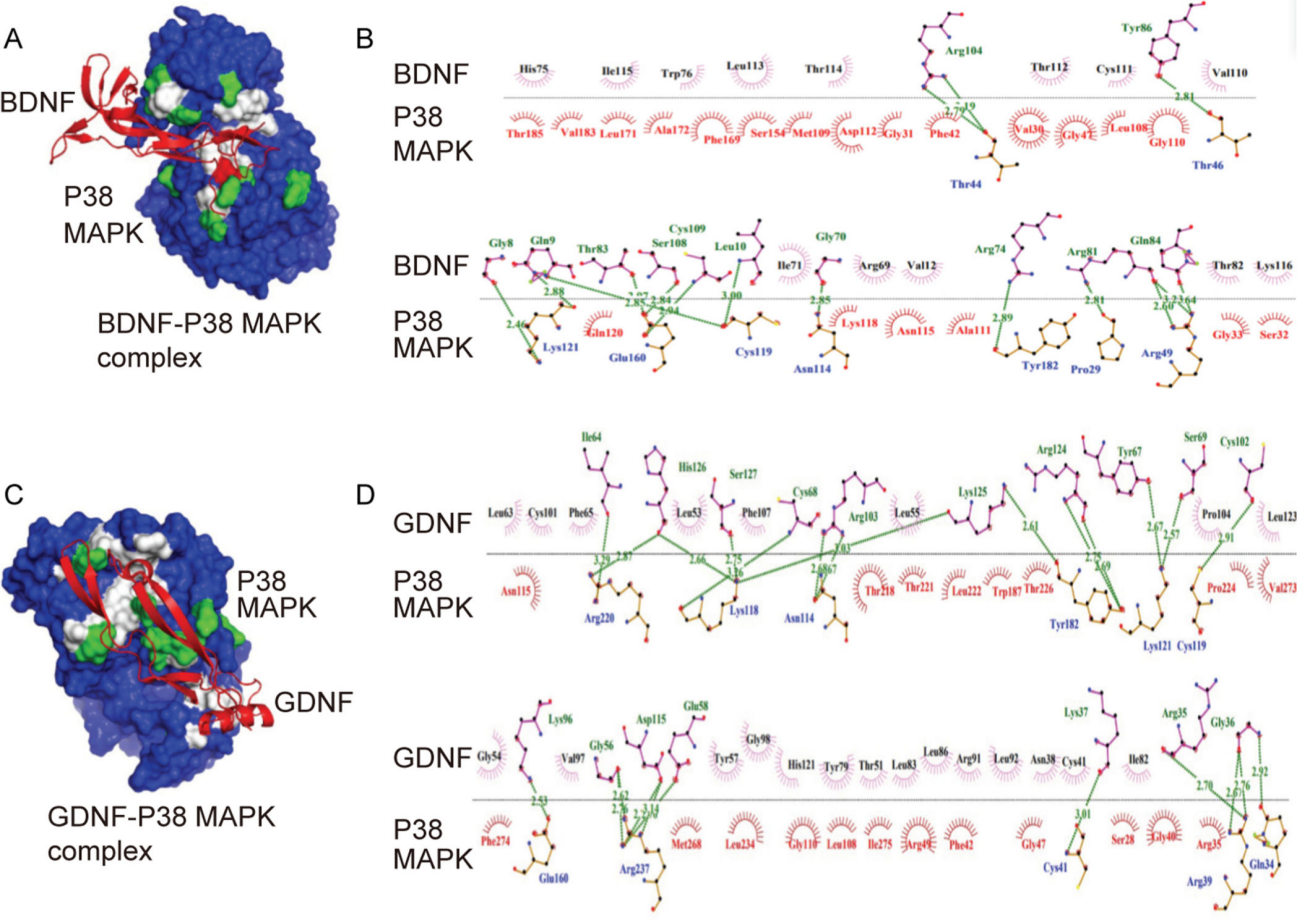
Interaction analysis of DPSC^℗^ BDNF, and GDNF with P38 MAPK. A. 3D surface model of the BDNF (red ribbon structure) bound with P38 MAPK (blue surface). B. 2D interaction pattern of the BDNF and P38 MAPK complex. C. 3D surface model of the GDNF (red ribbon structure) bound with P38 (blue surface). D. 2D interaction pattern of the GDNF and P38 MAPK complex. The green and white shade on the blue surface of P38 MAPK indicates the binding sites, where non-polar interactions (Van der Waals interaction) and polar interactions (hydrogen bonding) are denoted by white and green color, respectively. The blue-colored amino acid residues of P38 MAPK interacted with green-colored amino acid residues of BDNF/GDNF through hydrogen bonds. Van der Waals interaction occurs between the purple amino acid residues of BDNF/GDNF and the red amino acid residues of P38 MAPK.

**Fig. 8.**
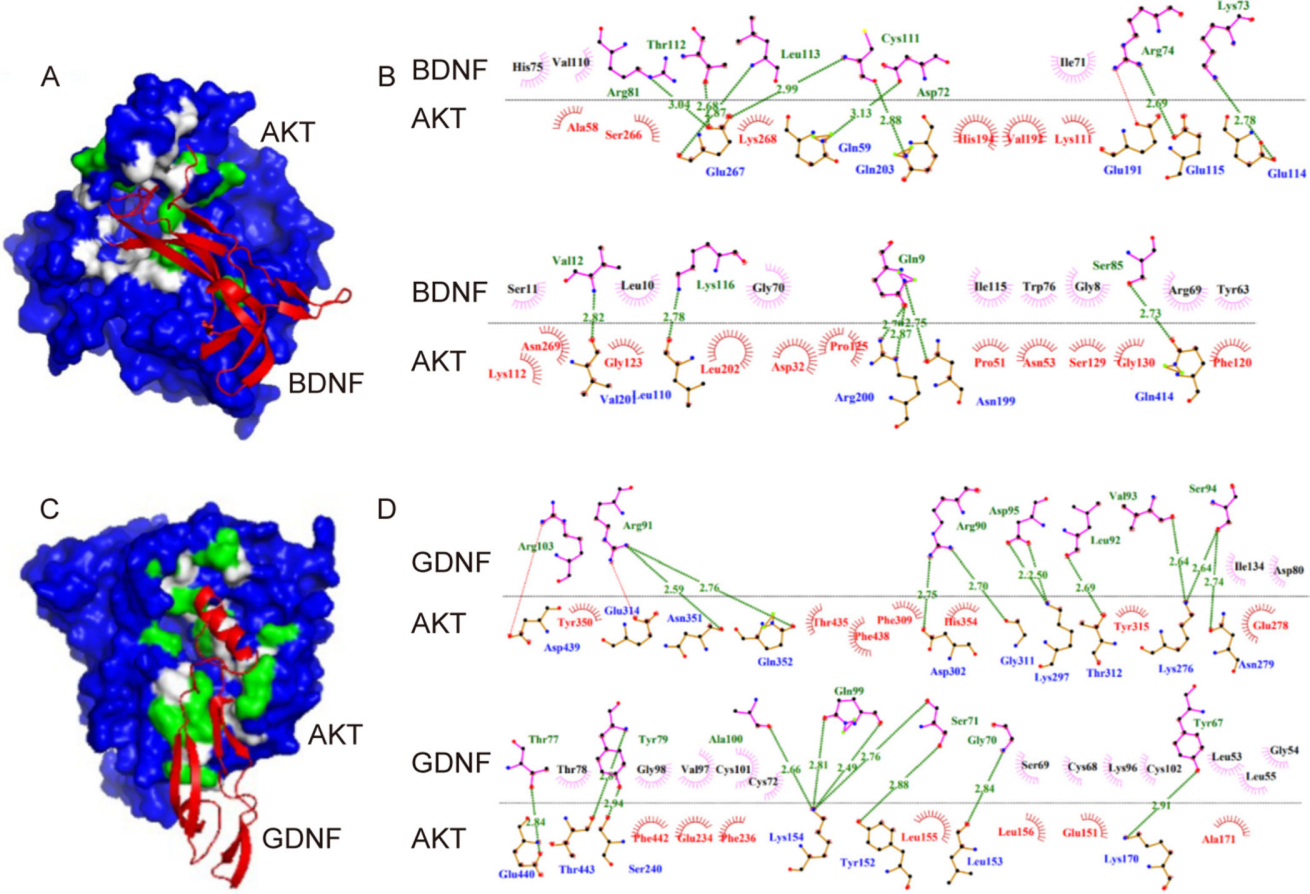
Interaction analysis of DPSC^℗^ BDNF and GDNF with AKT. A. 3D surface model of the BDNF (red ribbon structure) bound with AKT (blue surface). B. 2D interaction pattern of the BDNF and AKT complex. C. 3D surface model of the GDNF (red ribbon structure) bound with AKT (blue surface). D. 2D interaction pattern of the GDNF and AKT complex. The green and white shade on the blue surface of AKT indicates the binding sites, where non-polar interactions (Van der Waals interaction) and polar interactions (hydrogen bonding) are denoted by white and green color, respectively. The blue-colored amino acid residues of AKT interacted with green-colored amino acid residues of BDNF/GDNF through hydrogen bonds. Van der Waals interaction occurs between the purple amino acid residues of BDNF/GDNF and the red amino acid residues of AKT.

Our previous studies showed that DPSC^℗^ contains the growth factors, namely, brain-derived neurotropic factors (BDNF) and glial cell-derived neurotropic factors (GDNF), which are very relevant to the GBM cells. We hypothesized that these factors might combat the activation of the inflammatory pathway. Therefore, we took advantage of the system biology approach and determined the interaction between the 3D structures of BDNF, GDNF, and P38 MAPK using the ClusPro tool. The best interaction confirmation of BDNF and GDNF bound with P38 MAPK is represented in Fig. 7A, B. BDNF forms sixteen hydrogen bonds with the amino acids of P38 MAPK. In comparison, GDNF binds more strongly by forming twenty-four hydrogen bonds (Fig. 7C, D). The BDNF and P38 MAPK forms hydrogen bond between Arg104 and Thr44, Tyr86 and Thr46, Gly8 and Lys121, Gln9 and Lys121, Gln9 and Cys119, Thr83 and Glu160, Ser108 and Glu160, Leu10 and Cys119, Gly70 and Asn114, Arg74 and Tyr182, Arg81 and Pro29, and Gln84 and Arg49, respectively (Fig. 7B). The amino acid of p38 involved in hydrogen bonding with GDNF are Arg220, Lys118, Asn114, Glu160, Arg237, Tyr182, Lys121, Cys119, Arg39 and Gln34 (Fig. 7D). Interestingly, both BDNF and GDNF blocked the phosphorylation site (Tyr 182) of p38 and might hinder the activation of P38, resulting in the inhibition of downstream signaling. We also analyzed the interaction of BDNF and GDNF with AKT, which is shown in Fig. 8. We found that BDNF interacted with some pocket residues along with some other amino acids including Asp32, Pro51, Asn53, Ala58, Gln59, Glu191, Lys112, Glu114, Glu115, Phe120, Gly123, Pro125, Ser129, Gly130, Asn199, Arg200, Leu202, Gln203, Ser266, Glu267, Lys268, Asn269, and Gln414 by establishing fourteen hydrogen bonds and seventeen Van der Waals interactions (Fig. 8A, B). In contrast, GDNF binds more strongly by forming twenty hydrogen bonds and two salt-bridge (Fig. 8C, D). The GDNF and P38 MAPK forms hydrogen bond between Arg91 and Asn351, Arg91 and Gln352, Arg90 and Asp302, Arg90 and Gly311, Asp95 and Lys297, Leu92 and Thr312, Val93 and Lys276, Ser94 and Asn279, Ser94 and Lys276, Thr77 and Glu440, Tyr79 and Thr443, Tyr79 and Thr443, Ala100 and Lys154, Gln99 and Lys154, Ser71 and Lys154, Ser71 and Tyr152, Gly70 and Leu153, Tyr 67 and Lys170, respectively (Fig. 8C, D).

## Discussion

Inflammation plays a critical role in the sustained progression of tumors^[39]^. Chronic inflammation can elicit cancer cells’ growth and metastasis and contribute to treatment resistance^[40]^. Inhibition of inflammation can therefore help to slow down or prevent aggressiveness in cancer progression, improving the effectiveness of immune-based therapies. Harnessing MSC to modulate the immune tumor microenvironment has led to a breakthrough in ameliorating cancer progression. DPSC, one of the oral-derived MSC that were extensively investigated, exhibit a vast range of plasticity, including commitment to different lineages such as osteoblasts, adipocytes, chondrocytes, hepatocytes, myocytes, and neurons^[41]^. DPSC have shown remarkable therapeutic and regenerative efficacy for a wide range of pathological conditions due to their ability to secrete cytokines, growth factors, and extracellular matrix components. When the DPSC and DPSC^℗^ are injected into animal models of disease, they interact with the local cellular environment and exhibit paracrine actions resulting the cardioprotective, neuroprotective, and anti-fibrotic effects^[42]^. DPSC^℗^ has been suggested in promising therapeutics in the era of cell-free therapy^[42]^.

Tumors create a hostile niche that not only resembles a stem cell niche but also promotes tumor growth, malignancy, and resistance to the therapy. The brain tumor niche is composed of several specialized cell types, such as glioblastoma stem cells (GSC), tumor-associated macrophages, brain endothelial cells, and pericytes^[43]^. Each cell type contributes to shaping the tumor microenvironment and plays a pivotal role in tumor progression. Glioblastoma stem cells initiate tumor formation through mutations. Various types of scaffolds, both natural and synthetic, have been investigated for developing cancer therapy as they provide an ideal tissue microenvironment with histocompatibility and biosafety^[44]^. MSC^℗^, behaving like a natural scaffold, exert remarkable paracrine activity in tumor tissues, including the induction of tumor cell apoptosis, sensitization of tumors to chemotherapy, and repair of brain tissue damage^[45]^. Therefore, our study aimed to investigate the anti-inflammatory and anti-proliferative effect of DPSC^℗^ on human GBM cells, which is known to be aggressive cancer generally life span is 14 months to a year after diagnosis with GBM^[46]^. We first examined the anti-inflammatory and pro-inflammatory markers in U-87 MG cells in the absence and presence of DPSC^℗^. Our data strongly supported the previous studies^[13,47]^, representing DPSC^℗^ possess the ability to reduce inflammation by upregulating the expression of anti-inflammatory markers such as IL10, IL4R, and Arg1 and downregulating the expression of pro-inflammatory markers, including TNFα, IL1β, COX2, p65, MMP9, and IL6. Growing embodies showed that IL1, IL6, and TNFα are critical drivers of tumor growth, progression, and metastatic spread^[48]^. Thus, inhibition of inflammatory milieu by DPSC^℗^ may inhibit GBM by modulating the tumor-promoting factors.

The significance of ROS in the inflammatory response is well documented, as they are involved in initiating, progressing, and resolving inflammation. ROS acts as a second messenger in intracellular signaling and regulates several cellular phenomena, including cell proliferation and inflammation^[49]^. Excessive ROS is directly linked with cancer progression^[50]^. Therefore, we next examined whether DPSC^℗^ inhibits inflammation through ROS-mediated signaling. The confocal imaging analysis of DCFDA and MitoSOX revealed that DPSC^℗^ remarkably reduces the production of the total intracellular and mitochondrial ROS in GBM cells, suggesting that DPSC^℗^ obstructs excessive ROS production, which is a by-product of high metabolically active cells.

Mitochondria aids in sustaining the growth and survival of tumor cells by regulating the cellular energetics and metabolism. Mitochondria produces ATP by transferring electrons in the complex of the electron transport chain (ETC). These electrons are generated by oxidation of substrate; known as oxidative phosphorylation. The transfer of electrons from the inner space to the mitochondrial matrix generates a gradient of MMP^[51]^. Tumor cells demand high energy for expedited growth and proliferation; i.e. they produce high ATP, ultimately resulting in high MMP^[52,53]^. Therefore, we were interested in examining the MMP in GBM cells after the addition of DPSC^℗^. We found that the high MMP of GBM cells turned into low MMP after the addition of DPSC^℗^, suggesting that most of the GBM cells were metabolically inactive. These findings were corroborated with previous studies which reported chloroquine-induced GBM cell death is associated with mitochondrial membrane potential loss^[54]^. As MMP plays a critical role in mitochondrial bioenergetics, hence, we further investigated the effect of DPSC^℗^ on mitochondrial respiration and ATP production. We have performed OCR analysis using a flux analyzer and found that all the parameters of OCR, including ATP production, were decreased after the addition of DPSC^℗^ to the culture of GBM cells. Recent studies showed that the treatment with IACS-010759, an inhibitor of mitochondrial ETC robustly suppressed acute myeloid leukemia (AML) by depleting energy production through oxidative phosphorylation^[55]^. So, our findings suggested that DPSC^℗^ might have a similar ability to enforce metabolic reprogramming in GBM cells by disrupting oxidative phosphorylation.

Supporting evidence demonstrated that cancer cells rely not only on oxidative phosphorylation for the production of ATP but also on glycolysis for energy production, even in the presence of oxygen; this adaptation of tumor cells is known as the Warburg effect^[56]^. Thus, we also performed the ECAR analysis, and the obtained data were in favor of our expectation; all the parameters, including non-glycolytic acidification, glycolysis, glycolytic capacity, and glycolytic reserve were reduced in GBM cells after the addition of DPSC^℗^ during the culture of cells. These findings were aligned with the previous report, which demonstrated that treatment with trametinib in glioma cells significantly decreased the glycolytic activity and glycolytic reserve^[57]^. The obtained findings suggest that DPSC^℗^ has the ability to target cancer cell metabolism, which is a promising approach for developing novel cancer therapies. Based on the aforementioned findings and previous scientific evidence of the antineoplastic effects of MSCs^[20]^, we anticipated that DPSC^℗^ may have anti-proliferative effects on GBM cells; therefore, we performed the MTT assay to investigate the anti-proliferative effects on GBM cells. The results met our expectations and revealed that the addition of DPSC^℗^ for 24 h reduces the proliferation of GBM cells. Next, we were curious to investigate how DPSC^℗^ reduces the cell proliferation of aggressive GBM cells. The results showed that after the addition of DPSC^℗^ arrested the cell cycle at the G1 phase of the GBM cells. These findings were aligned with the previous study, which reported that the conditioned media from the umbilical cord and bone marrow-derived MSC (MSC-CM) halted the cell cycle of patient-derived GBM cells and increased the number of G1 phase population^[58]^. Subsequently, we wanted to understand in-depth molecular mechanisms of how DPSC^℗^ controls the inflammation and proliferation in GBM cells. To determine that we have taken advantage of the system biology approaches to catch clues of the DPSC^℗^ targeted pathway in GBM cells. We have reported earlier that DPSC secrets several neurotrophic factors, such as BDNF and GDNF, in the culture medium^[14]^. So, we established the interaction of the BDNF and GDNF with P38 MAPK and AKT using the clusPRO tool and found that both BDNF and GDNF clogged the phosphorylation site (Tyr 182) of P38 and might hinder the activation of the P38 MAPK cascade because phosphorylated tyrosine residue plays a crucial role to further trigger the downstream cascade^[59]^. MAPK pathway is activated by oxidative stress and mediates various cellular processes such as inflammation, cell cycle machinery, cell death, and differentiation^[60]^. So, inhibition of intracellular and mitochondrial ROS by DPSC^℗^ also reflects the deactivation of the P38 MAPK cascade. It is documented that targeting MAPK and AKT signaling pathways concomitantly is a promising approach for cancer treatment. Therefore, we also assessed the interaction analysis of BDNF and GDNF with AKT. BDNF and GDNF interact with a pocket residue of AKT but do not directly block the phosphorylation of Thr308 which is essential to the auto-phosphorylation of Ser473^[61]^. BDNF and GDNF might distort the catalytic pocket of AKT. To validate our computational data, we performed the western blot and found a significant decrease in the activation (phosphorylation) of P38 in GBM cells after the addition of DPSC^℗^. We were also interested in assessing whether blockade of the P38 MAPK pathway interplay with the PI3K/AKT pathway, which is crucial for cell survival and cell proliferation and found that after the addition of DPSC^℗^ also impedes activation (phosphorylation) of AKT in GBM cells. Previous studies have shown that the anti-proliferative effect of human umbilical cord-derived MSC against malignant glioma cell lines (SNB19 and U251) is associated with the downregulation of p-AKT. Our studies supported and corroborated these earlier reports. Furthermore, we provided the first evidence of the MAPK pathway is being targeted by DPSC^℗[62]^. Several studies demonstrated that the phosphorylation of checkpoint kinases, CHK1 and CHK2 triggers the downstream signaling to block the cell cycle progression. Most commonly, activation of CHK1 resulted in the G2/M phase of the cell cycle arrest by phosphorylation of Cdc52C^[63, 64]^. However, several pieces of evidence suggested that CHK1 activation also involves in the G1 phase cell cycle, and targeting the CHK1 for developing potential anticancer regimes is considered an excellent approach^[63]^. Here, we found that DPSC^℗^ activates the phosphorylation of CHK1 in GBM cells. These studies were corroborated by previous studies showing the activation of CHK1 by piperine arrests the cell cycle at the G1 phase in melanoma cells^[64]^. Overall, DPSC^℗^ exerts anti-proliferative and anti-inflammatory effects by interfering in the P38 MAPK-AKT signaling pathway.

## Conclusion

The current study highlights the significance of DPSC^℗^ in combating the aggressiveness of GBM cells. We found that DPSC^℗^ remarkably inhibits the inflammation and proliferation of GBM cells by suppressing the cancer cells’ metabolism and arresting the cell cycle at the G1 phase. We also pointed out that DPSC^℗^ inhibits the activation of P38 MAPK and AKT and induces the phosphorylation of the checkpoint molecule CHK1. Taken together, DPSC^℗^ could be a promising, non-invasive approach for combatting tumor aggressiveness with minor/no adverse effects. Additionally, MSC^℗^ exhibit ability to sensitize the tumor cells as reported previously; DPSC^℗^ may be used as an adjuvant therapy in combination with other treatment modalities, such as chemo or radiation therapy in clinical studies. However, further research is warranted to fully comprehend the therapeutic potential of DPSC^℗^ for regulating GBM growth, and their safety and efficacy in *vivo* models.

## Supplementary Material

Supplementary

## Data Availability

The data that support the findings of this study are available from the corresponding author upon reasonable request.

## Abbreviations

AKT: Protein kinase B
Ala: Alanine
AML: Acute myeloid leukemia
Arg: Arginine
Arg1: Arginase 1
Asn: Asparagine
Asp: Aspartic acid
BDNF: Brain-derived neurotrophic factor
CHK1: Checkpoint kinase 1
CHK2: Checkpoint kinase 2
CNS: Central nervous system
COX2: Cyclooxygenase 2
Cys: Cysteine
DAPI: 4’,6-diamidino-2-phenylindole
DCFDA: Dichlorodihydrofluorescein diacetate
DPSC: Dental pulp-derived stem cells
DPSC^℗^: DPSC secretome
ECAR: Extracellular acidification rate
ETC: Electron transport chain
GBM: Glioblastoma
GDNF: Glial cell-derived neurotrophic factor
Gln: Glutamine
Glu: Glutamate
Gly: Glycine
HBSS: Hanks’ balanced salt solution
His: Histidine
IL10: Interleukin 10
IL1β: Interleukin 1β
IL4R: Interleukin 4 receptor
IL6: Interleukin 6
Ile: Isoleucine
JC1: Mitochondrial membrane potential probe
Leu: Leucine
lncRNA: long non-coding RNA
Lys: Lysine
MEME: Minimum essential medium eagle
Met: Methionine
miRNA: microRNA
MitoSOX: Mitochondrial superoxide indicator dye
MMP: Mitochondrial membrane potential
MMP9: Matrix metallopeptidase 9
MSCs: Mesenchymal stem cells
OCR: Oxygen consumption rate
P38 MAPK: Mitogen-activated protein kinases (P38)
P65: Nuclear factor kappa B subunit P65
PDB: protein data bank
Phe: Phenylalanine
Pro: Proline
qRT-PCR: Quantitative reverse transcription-polymerase chain reaction
ROS: Reactive oxygen species
Ser: Serine
siRNA: small interfering RNA
TBS: Tris-buffered saline
TNFα: Tumor necrosis factor α
Trp: Tryptophan
Tyr: Tyrosine
Val: Valine
