# Supplementary material for: Secretome of Dental Pulp-Derived Stem Cells Reduces Inflammation and Proliferation of Glioblastoma Cells by Deactivating Mapk-Akt Pathway
Source: Dis Res. Author manuscript; Available in PMC 2024 Jan 11. (PMC10783424; doi:10.54457/DR.202302006)
Supplement: Supplementary [file NIHMS1918879-supplement-Supplementary.pdf]

Supplementary Figures

Secretome of dental pulp-derived stem cells reduces inflammation and proliferation of glioblastoma cells by deactivating MAPK-AKT pathway

Prateeksha Prateeksha[§], Md Sariful Islam Howlader[§], Surajit Hansda[§], Prathyusha Naidu, Manjusri Das, Faten Abo-Aziza and Hiranmoy Das[*]

Department of Pharmaceutical Sciences, Jerry H. Hodge School of Pharmacy,
Texas Tech University Health Sciences Center, Amarillo, Texas, USA.

§ Equally contributed.

Supplementary Figure 1

List of gene and their primer sequences

No	Gene	Forward	Reverse
1	**βActin**	5′-TGG ACT TCG AGC AAG AGA TG -3′	5′-GAA GGA AGG CTG GAA GAG TG-3′
2	**COX2**	5′-TTC AAA TGA GAT TGT GGG AAA ATT GCT-3′	5′-AGA TCA TCT CTG CCT GAG TAT CTT-3′
3	**IL6**	5′-AGA CAG CCA CTC ACC TCT TCA G-3′	5′-TTC TGC CAG TGC CTC TTT GCT G-3′
4	**MMP9**	5′-GCC ACT ACT GTG CCT TTG AGT C-3′	5′-CCC TCA GAG AAT CGC CAG TAC T-3′
5	**p65**	5′- TGAACCGAAACTCTGGCAGCTG-3′	5′- CATCAGCTTGCGAAAAGGAGCC-3′
6	**TNFα**	5′- CTCTTCTGCCTGCTGCACTTTG-3′	5′-ATGGGCTACAGGCTTGTCACTC-3′
7	**Arg1**	5′-TCATCTGGGTGGATGCTCACAC-3′	5′-GAGAATCCTGGCACATCGGGAA-3′
8	**IL1β**	5′-CCACAGACCTTCCAGGAGAATG-3′	5′-GTGCAGTTCAGTGATCGTACAGG-3′
9	**IL4R**	5′-CTGCTCATGGATGACGTGGTCA-3′	5′-GGTGTGAACTGTCAGGTTTCCTG-3′
10	**IL10**	5′-TCTCCGAGATGCCTTCAGCAGA-3′	5′-TCAGACAAGGCTTGGCAACCCA-3′

Supplementary Figure 1. Primer sequences used for qRT-PCR studies.

Supplementary Figure 2

Primary antibody used for western blot analysis

No	Antibody and their Catalogue No.	Company	Dilutions for Western Blot	Host
1	**pAKT #2965S**	Cell signaling	1:1000	Rabbit
2	**AKT #2920S**	Cell signaling	1:1000	Rabbit
3	**pP38 #9211S**	Cell signaling	1:1000	Rabbit
4	**P38 #9212S**	Cell signaling	1:1000	Rabbit
5	**pCHK1 #2347S**	Cell signaling	1:1000	Mouse
6	**CHK1 #2360S**	Cell signaling	1:1000	Rabbit
7	**GAPDH #2118L**	Cell signaling	1:1000	Rabbit

Supplementary Figure 2. Primary antibody details used for western blot studies.

Supplementary Figure 3

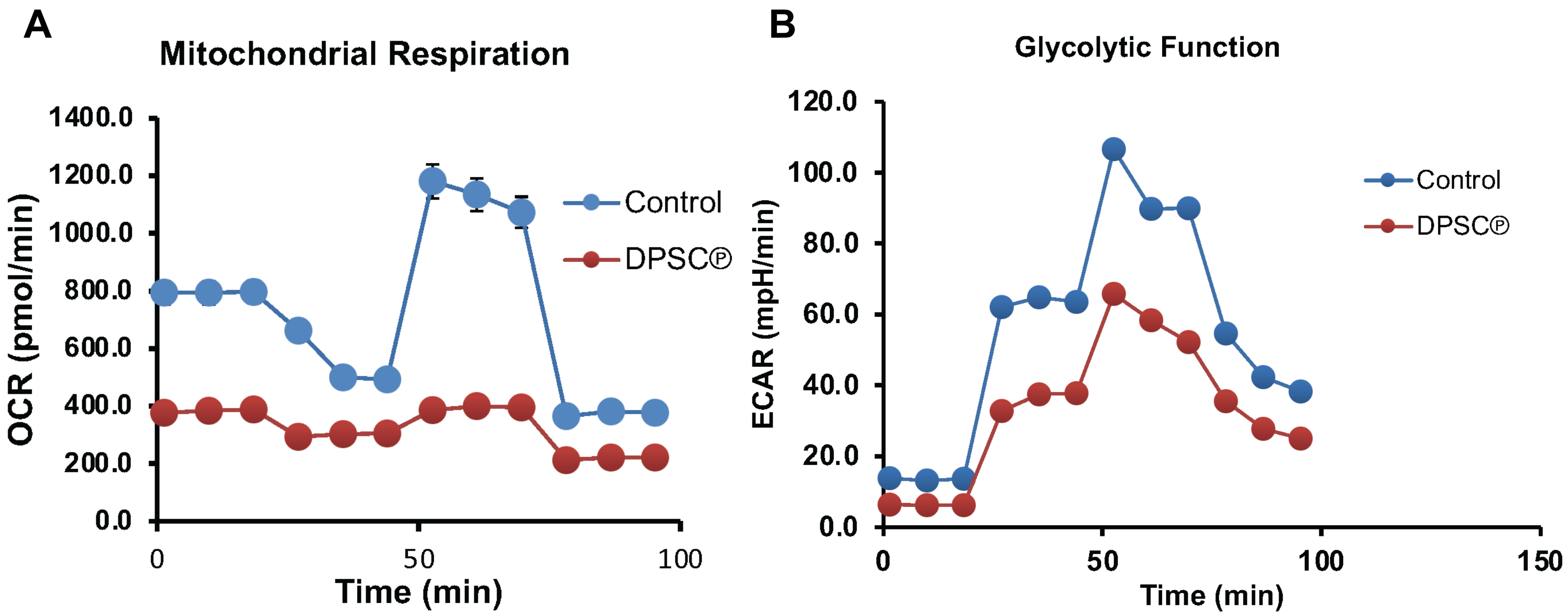

Supplementary Figure 3. A. Showing original seahorse extracellular flux analysis graph of oxygen consumption rate (OCR) in glioblastoma cells in U-87 MG cells in the presence or absence of DPSC℗ during the culture of cells for 24 h. **B.** Showing original Seahorse extracellular flux analysis graph of extracellular acidification rate (ECAR) in in U-87 MG cells in the presence or absence of DPSC℗ during the culture of cells for 24 h.

Supplementary Figure 4

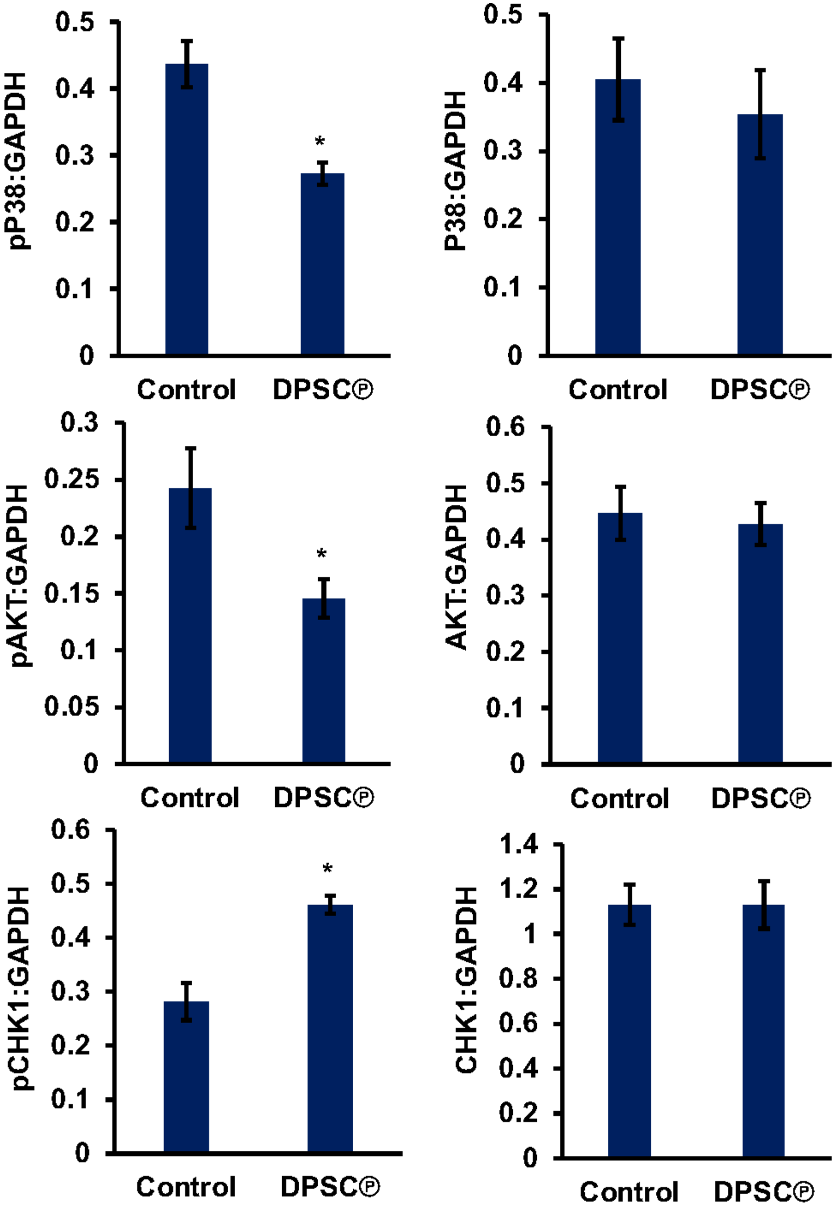

Supplementary Figure 4. Bar graphs represent the quantified level of pP38, P38, pAKT, AKT, pCHK1, and CHK1 proteins corresponding to GAPDH shown in original Figure 6 in the manuscript.
